# The Unseen Costs of Low-Income Work: Understanding the Relationship Between Parent Work and Child Cognitive and Academic Outcomes

**DOI:** 10.3390/bs16071089

**Published:** 2026-07-02

**Authors:** Mark D. Agars, Gino J. Howard

**Affiliations:** Department of Psychology, Institute for Child Development and Family Relations, California State University, San Bernardino, CA 92407, USA; gino.howard@csusb.edu

**Keywords:** child school outcomes, work-family interface, spillover-crossover model, low-income parents

## Abstract

Parents play a central role in their child’s school outcomes. For working parents, however, particularly those working low-wage jobs, managing work and family demands is a constant and often overwhelming reality that can have significant and adverse effects on engagement with family and children. Much of our understanding of the relationship between parental work and child school outcomes, however, has been limited to broad contextual factors (e.g., income level, time). As articulated through Bioecological theory, the context through which children’s school outcomes may be influenced is layered and multifactorial. The Job Demands-Resources model and spillover-crossover model of the work-family interface provide a theoretical lens through which we can examine how direct work factors, as well as parental efforts to navigate the work-family interface, impact child school outcomes. The purpose of this paper is to explicate the integration of Bioecological theory and Spillover-crossover theory to provide a framework for examining these factors and to highlight several areas in the work-family literature that are ripe for exploration of their role in child school outcomes. For children of parents working low-wage jobs, the detrimental effects of parent work factors on parent–child interactions and child outcomes are particularly salient. By leveraging work-family theory and established literature on parental involvement, this review provides an interdisciplinary approach to understanding the link between the systems that shape parent experiences (i.e., their work roles) and child cognitive and academic outcomes.

## 1. Introduction

Growing evidence demonstrates the detrimental consequences for children when parents work low-wage jobs (Fuller et al., 2025; Walther & Pilarz, 2024), yet the nature of this relationship is not well understood. More broadly, for children living in poverty, the negative impact on cognitive development and school outcomes is well documented (Bradley & Corwyn, 2002; Gibson-Davis et al., 2022), with effects only worsening with sustained exposure (Suntheimer & Wolf, 2024). Children living in low-income families display deficits relative to children of middle- and high-income families in executive function (Hackman et al., 2015) and school achievement (Duncan et al., 2011). These deficits are a function of the broader developmental context and the impact living in poverty has on children. Given the centrality of parent work to family economic status, the overwhelming majority of children and youth living in low Socioeconomic status (SES) households have parents or caregivers working precarious, low-wage jobs. Moreover, the prevalence of low-wage work in the U.S. (21% of workers—31 million people earn $17 per hour or less, Economic Policy Institute, 2026) is significant and growing. Providing a path for research to better understand the role of parent work factors in contributing to this developmental context, and to child cognitive and school outcomes more specifically, is the goal of this paper.

We believe that explicating the impact of low-wage parental work, beyond direct income effects, offers fruitful paths for expanding our understanding of the SES—child academic outcomes relationship. Low-wage jobs typically operate on non-standard schedules, while providing employees with little control and few resources for managing work and family responsibilities (Agars & French, 2016; Lambert et al., 2019). Low-wage positions often include uncertainty around work availability, unpredictable hours, and few organizational supports for managing competing work and family demands (Kossek & Lautsch, 2018). Although schedule flexibility is typically considered a tool for workers to manage their work and family responsibilities, the opposite is true when flexibility is non-voluntary (Kaduk et al., 2019). Moreover, the lack of predictable schedules for low-wage workers undermines perceptions of security (Lambert et al., 2019), which can have damaging effects on parental mental health and family environment (Walther & Pilarz, 2024). In short, parent work characteristics beyond income are likely but underexplored factors shaping cognitive and school outcomes for their children. Many of these factors (e.g., limited resources, flexibility, and schedule control) are shared among those who are part of low-income, SES, and wage groups. This highlights that while further exploration of each group is warranted, the shared experiences provide common ground to explore how parent work factors similarly impact parent–child relations and child outcomes in each of these groups.

Research into the work-family interface includes scholars from a breadth of disciplines. Despite this broad interest, as is often true of research addressing phenomena that manifest across life domains, efforts too often fail to meaningfully capture the depth and richness of factors across domains simultaneously, tending instead to be deep in one, but shallow in the other; more singularly focused on domain-specific relationships, while ignoring or controlling extra-domain factors. Research in the industrial-organizational psychological realm, for example, routinely captures the impact of work factors on employee mental health (Hammer et al., 2021; Richardson, 2017), but too often fails to further consider the impact on the broader social network or family system, such as the relationships between employed parents and their children. Similarly, child development research aimed at better understanding parental work effects on children often examines blunt work factors such as time (Hsin & Felfe, 2014; Kalil & Ziol-Guest, 2008) and income (Rakesh et al., 2025), with limited consideration of the nuanced work experiences and their paths of influence. There are exceptions (e.g., Fuller et al., 2025; McCredie et al., 2025), though their contributions serve to highlight the need for integrated multidisciplinary approaches. To encourage and guide this work, we draw on our organizational psychology background to illustrate how work-family theory may inform a systems-based approach to understanding how parent work factors impact child cognitive development and school outcomes, particularly for children of parents working low-wage jobs.

## 2. Necessity of a Systems Model

Although research on the impact of parent work characteristics and work-family experiences on child development has advanced in recent years, it remains limited in its consideration of the processes through which work factors and work-family experiences shape child cognitive and academic outcomes (Cho & Ciancetta, 2016; Orr & Caspi, 2025). Moreover, research examining the work-family interface has rarely considered the sophistication of both work domain and family domain factors simultaneously. These limitations are particularly relevant for low-wage workers (Agars & French, 2016; Bruns et al., 2025; French & Agars, 2018), who often face the greatest challenges managing the work–life interface, while in positions that offer the least support (Kossek & Lautsch, 2018; Swanberg et al., 2014).

Much of the research relevant to low-wage workers and parenting has utilized the lens of non-standard work, broadly demonstrating a negative effect on child cognitive outcomes (Han, 2005), though with little consideration of explanatory mechanisms. Subsequent work identified potential explanations, including Grzywacz et al. (2011), who found that non-standard work schedules were negatively related to maternal sensitivity and home environment. Additionally, Haines et al. (2020) found that non-standard work can impact parenting quality through experiences of work-family conflict and strain. Non-standard work can directly impact parent stress because it contributes to income insecurity, and inconsistent parent–child interaction time and space. These studies are important illustrations of the impact non-standard work can have on parenting and, ultimately, children, as downstream effects of limited resources available to low-wage parents. Therefore, an expanded consideration of work and family domain characteristics specific to low-wage workers is needed. Interviews with women working low-wage jobs reveal that “flexibility” associated with low-wage jobs is often perceived as causing unpredictability, being tied to insufficient hours, and used by leaders as a tool for punishment and control rather than support (Jacobs & Padavic, 2015). What is the impact of these perceptions on working parents in their family roles?

The lack of flexibility and control for low-income parents may manifest as higher stress levels (Nomaguchi & Johnson, 2016), highlighted by the stress–strain model (Bolger et al., 1989; Koeske & Koeske, 1993). While increased flexibility and control are resources parents utilize to plan family commitments and parent–child interactions, their absence creates inconsistency and perceptions of unreliability, which may strain relationships and increase stress. Experiencing stress that is relatively low and short-term does not typically have long-term effects, but encountering many stressors, or long-term stress, a more common experience among low-wage workers, is related to poorer psychological and physical health (Ford et al., 2014; Mazzola & Disselhorst, 2019). Parenting stress may manifest from experiences, expectations, or needs from the parent and/or the child (Crnic et al., 2005), and contribute to lowered energy, worsened mood (Demerouti et al., 2001; Maslach, 2003), and the inability to fulfill their parent role (Dunning & Giallo, 2012).

**Figure 1:**
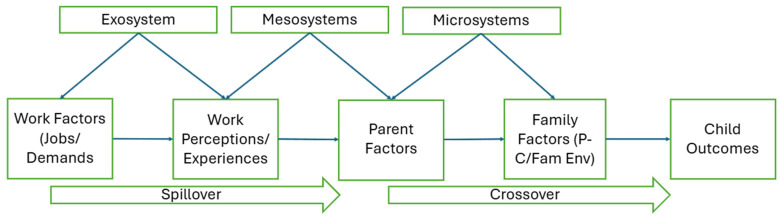
Proposed multidisciplinary process model. Note. P-C = parent–child; Fam Env = family environment.

These stressful experiences from work-related factors have downstream effects on parents and their children. Though the centrality of parents to child development is well established, the process has yet to be addressed from a multilevel and multidisciplinary perspective. We propose a multi-level systems model, grounded in Bronfenbrenner’s Bioecological theory (1994), while integrating work-family theories to explicate more precise explanatory processes (Figure 1).

## 3. Multiple Theories with a Singular Focus: Building Out a Process Model

Considering the complexity of paths through which work-related factors impact working parents, the home environment, and their children, an integrated theoretical approach provides an ideal framework. Ecological systems theory (Bronfenbrenner, 1994; 2005), role theory (Biddle, 1986; Greenhaus & Beutell, 1985), Job Demands-Resources theory (Bakker & Demerouti, 2007), and the spillover-crossover model (Bakker & Demerouti, 2018), provide a theoretical foundation for examining the direct, indirect, and bidirectional relationships between work factors, family and parent factors, parent–child interactions, and child cognitive and academic outcomes.

According to Bronfenbrenner’s (1994) Bioecological theory of development, children develop within the context of a set of systems that exert direct and/or indirect influence on their developmental experience. The child’s immediate environment consists of microsystems with which a child engages directly. Critical among these are parent–child interactions and the broader family environment. These proximal environments are influenced by more distal exosystem factors such as the parents’ work environment. Although not directly experienced by the child, exosystem factors can have both positive and negative effects on their development through indirect influences. Mesosystems refer to the places and processes through which exosystems and microsystems interact. In the context of the work-family interface, these systems and their interactions are central to understanding the impact of parent work on child cognitive and academic outcomes and offer an ideal opportunity to integrate work-family theory.

Parental work represents an external system (exosystem) that affects child development indirectly through its impact on parent outcomes and parent–child interactions (Cho & Ciancetta, 2016). The Job Demands-Resources model (Bakker & Demerouti, 2007) explains how parental work demands (e.g., workload, emotional strain, unpredictable hours) can disrupt the potential for healthy interactions between parents and their children, especially for low-wage families (Heinrich, 2014). Conversely, workplace resources such as autonomy and support provide opportunities for parents to approach interactions within the family system with energy and expectations needed to buffer work demands (Skinner & Ichii, 2015). As further articulated by work-family role theory (Greenhaus & Beutell, 1985; Biddle, 1986), working adults are constantly managing conflicting demands from both work and family domains, and challenges meeting those demands in either domain have emotional, behavioral, and cognitive consequences, especially for working parents and their children. The extent to which parents (in)effectively manage their roles and responsibilities impacts their relationships in each domain and, specifically, parent–child relationships, through positive and negative spillover and crossover (Bakker & Demerouti, 2018).

Family environment, parent characteristics, and parent–child interactions are each part of the immediate environment within which child development occurs and within which exists the potential for direct and reciprocal effects between parent and child (Merçon-Vargas et al., 2020). Spillover-crossover theory (Bakker & Demerouti, 2018) argues for both within-person (spillover) and between-persons (crossover) processes, which provide a framework for the indirect effects of work factors on child academic and cognitive outcomes. Specifically, spillover-crossover explains how emotions, behaviors, and stress that emerge in one domain (e.g., parent work) can transfer to another (e.g., family) and ultimately manifest in others who share the family domain (e.g., the child). Indeed, a growing body of evidence shows that parent workplace experiences impact child outcomes (Cho & Ciancetta, 2016) and spillover into home environments (Repetti & Wang, 2010), impact daily parent–child interactions (Sears et al., 2016) and spousal relationships (Repetti & Wang, 2017).^1^

Although each theory has traditionally been applied independently for different aspects of our proposed model, we might also consider the different ways in which combinations of these theories integrate, interact, and complement each other in predicting different systems and levels of interactions. For instance, parent work factors can be best examined using the JD-R model and subsequent evaluations of the parent work experience through role theory. Stress that manifests via demands, a lack of resources, and the parent appraisal process may spill over into the family domain and ultimately cross over to their children. This process happens through nested systems through which Bioecological theory explains the impacts on child development as shaped by the surrounding systems (i.e., child characteristics, family characteristics, and policy). In exploring these, there will be overlap between the theories that should be further addressed in future research. For example, parents’ experiences of stress could be categorized as a mismatch between demands and resources, role strain, negative spillover, or even the effects of the exosystem. We propose this model and integration of multiple relevant theories to highlight the importance of the multifaceted nature of these effects as opposed to presenting them as competing theories. The extent to which a specific combination theory is applied to future research questions and hypotheses must be further addressed and transparently reported in subsequent research.

Our goal is to provide guidance for scholarship aiming to understand how the parent–child interactions and the family environment (microsystems) that directly impact child cognitive outcomes, are impacted by parent workplace factors (exosystem), and parent experiences in managing the interaction between domains (mesosystem). Accordingly, our model argues that workplace demand and resource factors (exosystem) shape working parent experiences and perceptions of stress, well-being, and motivation, which, in turn, spill over into the family domain (mesosystem processes) and manifest as parent factors. These varying levels of parental stress, motivation, and emotion then directly impact the development of the family environment and parent–child interactions (microsystems), which assert direct influence on child development. The research on parental influence on child development, specifically cognitive development and school outcomes, is immense and clear in the vital role of parents. We aim to build out these findings by positioning them within the broader context of parent work and integrating insights from the work-family literature.

## 4. Impact of Low-Wage Work on Parents and Parenting

Low-wage employment typically combines low pay, unstable hours, lack of autonomy, and limited benefits (Van Aerden et al., 2014; Vanroelen, 2019). Consistent with role theory (Biddle, 1986; Greenhaus & Beutell, 1985), those jobs therefore create more time- and strain-based conflict because low-wage parents have fewer resources to substitute their own time and labor in the home/family domain. Strain transfers across domains for the working parent and spreads throughout the family system and into the development of children through spillover and crossover effects. With greater demands and limited resources, the experiences of spillover and crossover are more likely to occur and to be more extreme for more at-risk populations, such as low-wage workers (Bakker & Demerouti, 2007). A last-minute shift change, for example, can quickly become a childcare emergency, transportation problem, bedtime routine disruption, loss of homework help, or chaotic home routine. Compounding these challenges, low-wage positions typically have fewer (or completely lack) formal work–life management policies (Kossek & Lautsch, 2018), provide less discretion over worktime and place (Swanberg et al., 2011; Swanberg et al., 2014), and compensate less, all of which create barriers to finding alternative accommodations or supports. Working-class parents may also find it particularly difficult to both ask for and receive time off for family responsibilities, a reality that is particularly problematic for fathers (Nomaguchi & Johnson, 2016). Even models of supervisory support are based on traditional white-collar professional positions (French & Agars, 2018; Guo et al., 2024) and do not apply well to low-wage workers.

Because of the limited consideration of low-wage workers in research, even seemingly well-founded recommendations are not straightforward. Though research directs parents to be more involved with their children and to focus on high-quality interactions (Moroni et al., 2015), for low-wage working parents, this is inconsistent with their most commonly stated need, which is greater financial resources to support their family, which is most typically obtained by taking on additional work (Albelda & Shea, 2010). Moreover, this disconnect is further reflected in self-perceptions of low-wage working mothers, who report strong beliefs that they are contributing to their parental role when they take on extra work and earn more money for the family, even though it means less time with their child (Hennessy, 2009). Similarly, though common recommendations to workers in poverty focus on working additional hours to increase personal financial resources (Albelda, 2011) or increasing career potential by taking on more responsibility or going back to school, these solutions can be inconsistent with the needs of low-wage working parents. McManus and Suizzo (2021), for example, found that mothers living in poverty expressed a need for more time, money, and access to resources for their own self-care. While more parent education and specific training may be fruitful (McManus & Suizzo, 2021), the full potential may be lost when basic needs are not met. In sum, the quality of parent–child relations is a product of organizations and the system of work, and for parents working low-wage jobs, the challenges are severe and not well understood because of limited consideration in our research.

## 5. Parent–Child Relations and Child Outcomes

Time and energy are finite resources that parents devote to their work and family. When parents experience work-family conflict (Greenhaus & Beutell, 1985), they are drained of their energy, and their work and family roles are likely to suffer. Lower parental Socioeconomic status (SES) has been associated with a wide range of negative cognitive, language, and school outcomes in children and youth (Rakesh et al., 2025; Yeung et al., 2022), and low-SES contexts are inherently stressful and create lower quality parent–child interactions (Perzow et al., 2018). However, a recent meta-analysis (Song et al., 2025) noted that the relationship between SES and child educational outcomes is reduced significantly or even eliminated when other parent and family environment factors are controlled for. They argue for a more sophisticated theoretical approach to understand how SES factors shape child educational outcomes. Although deficits in child cognitive and school outcomes are often framed in the context of income, growing evidence suggests that related parental, familial, and environmental factors play a more critical role. As such, the quality and frequency of parent–child relationships may function differently and reciprocally due to parents’ experiences and characteristics of their child(ren). For instance, if a child has a particularly difficult temperament or is in a more difficult developmental stage, parents may experience more demands with the same limited resources. As we discuss different child outcomes below, we acknowledge that the relationships we highlight matter for both cognitive and non-cognitive outcomes. Even with consideration of individual parent and child characteristics, part of the answer is likely tied to characteristics of parents working low-wage jobs and their impact on how parents interact with their children. Though we touch on a range of child outcomes related to academic success (i.e., academic, behavioral, emotional, and cognitive), we note that the individual paths of influence likely include differences that should be explored along with the known interconnectedness of the outcomes themselves. In the subsequent sections, we touch briefly on how parents impact child outcomes related to school success, highlighting examples of how work factors matter. Subsequently, we provide a broader articulation of how the integration of work and family research and theory may serve as a valuable tool for advancing this work.

### 5.1. Academic Outcomes

When parents are involved in their child’s schooling, child engagement and school achievement improve (Bryce et al., 2019). Variability in parent engagement with their children also shifts during different developmental phases for children. As such, parent involvement at the preschool age (Ferreira et al., 2018) and middle and high school-aged children (Costa et al., 2024) relates to more academic success. Long-term outcomes for children’s academic success improve when parents and children have higher quality relationships characterized by emotional support and higher parental expectations for their children (Jeynes, 2024; Yamamoto & Holloway, 2010).

Holmes et al. (2018) demonstrated that work and family factors (in the form of full- vs. part-time employment status and work-family conflict) negatively impacted child academic achievement through less maternal school involvement. While parental involvement is beneficial for children’s cognitive and academic outcomes, low-wage parents are less likely to have access to resources that make more involvement a lived reality.

### 5.2. Behavioral and Emotional Outcomes

When parents engage more with their children in their early school years, children exhibit more self-control (Ferreira et al., 2018), social-emotional skills, and emotional regulation (Cosso et al., 2022). Yang et al. (2024) also found that secure parent–child attachment is related to more child academic engagement and motivation. Ferreira et al. (2018) showed, however, that for dual earner couples, both mothers and fathers had fewer developmental interactions with their children when they experienced higher levels of work-family conflict. Vahedi et al. (2018) demonstrated spillover and crossover effects of work factors on child and family outcomes, as work-family conflict was related to problem internalizing for children as well as inter-parental conflict. Some work-family research has demonstrated that the conditions of parent work through engagement may be (indirectly) related to child behavioral and emotional outcomes. When parents experience stress, they tend to engage in harsher parenting strategies that result in more problem internalizing and poorer social behavior for children (Negi & Sattler, 2025). Vieira et al. (2016) found further evidence that the parent–child relationship mediates the relationship between work-family conflict (spillover) and both internalized and externalized problem behaviors for children (crossover).

### 5.3. Child Cognitive Outcomes

Parents have a primary role in the cognitive development of children. Critical factors include parental sensitivity (Rodrigues et al., 2021), parent–child attachment (Deneault et al., 2023), and parental involvement (Barger et al., 2019). Parent work factors, especially for low-wage workers, matter for these relationships as well. Pilkauskas et al. (2018) found that maternal employment in low-income families was positively related to child well-being and cognitive skills, and the nature of the relationships is even stronger when work was stable and of higher quality. Most critically, that employment itself was positively related to child cognitive outcomes underscores the challenges of studying work factors for low-wage workers. Some factors (e.g., employment and hours) may serve as both a family demand (increasing work and family conflict) and a source of work and family enrichment (i.e., financial security). Indeed, family income gains for children in poverty are associated with later childhood cognitive functioning (Raffington et al., 2018). Parent stress is related to both their own behaviors and the quality of relationships with their children, highlighting how work-related stress may crossover from parent to child (Crnic et al., 2005). Zhang et al. (2025) found that when parent income was unstable or chronically low, children’s mental health worsened (i.e., higher rates of anxiety and depression), which subsequently impacts cognitive and school outcomes (Devine et al., 2025).

The culmination of these research findings shows the impact that parent–child interactions have on child outcomes socially, emotionally, and academically across developmental stages. When these findings and perspectives are combined with bioecological systems theory, factors that hinder (e.g., work-family conflict) or help (e.g., schedule predictability) parental involvement at the micro- and meso-level provide insight into how parental workplace factors permeate into child-level outcomes. This evidence also underscores how stability in parents’ and children’s lives, where resources are available for positive, engaging family environments and supportive work lives, supports child well-being and academic success. While finding resources and availability are critical for working parents in supporting their child’s development, there may be a harsher lived reality for parents working low-wage jobs. As such, it is critical to identify ways in which we can improve the process from parent work to child cognitive and academic outcomes.

## 6. Lighting the Path

### 6.1. Integrating Work-Family Theory

By overlaying work-family theories onto Bronfenbrenner’s model, our proposed systems model elucidates a path to advance understanding of how work factors matter for child cognitive and school outcomes by considering how micro-level theory and research in the work-family domain may be utilized. Examples are evident and growing. The challenge for low-wage working parents is that low-wage job characteristics are anathema to the development of family routines. Specifically, the overwhelming majority of low-wage jobs include schedule unpredictability, fluctuation in the number, or workable hours week to week, and non-standard (i.e., off-hours) work (Henly & Lambert, 2014). Further, work and family roles are more overlapping and with blurred boundaries, especially for low-wage working moms (Kuang et al., 2025). Each of these factors makes routine development difficult. As family routines are microsystem factors that influence cognitive and school outcomes for children, the impediment that low-wage work factors pose to their development needs to be better understood.

How can low-wage work roles include more stable resources such as cooperative scheduling, flexibility, and family support (Ananat & Gassman-Pines, 2021)? In what ways can organizations reduce demands by improving role clarity, job security, and creating a culture of inclusivity and respect (Sonnentag et al., 2023)? Most critically, how and in what ways might these changes impact children’s cognitive and school outcomes? It is important to note, as well, that exploring the work–life interface for low-wage workers is further complicated because low-wage work often intersects with demographic factors, including immigration status, single-parenting, and race (Agars & French, 2016). Attention must be paid to unpacking the independent and integrative roles each plays. Likewise, the work-family literature has long been limited in its inclusivity of working populations (c.f., Agars & French, 2016). Therefore, in addition to the solutions presented below, future research must more directly include fathers, workers of color, non-standard workers, the formerly incarcerated, and LGBTQ+ identified workers, among other historically excluded populations, to capture the breadth and nuance of low-wage family systems. Finally, our work must also consider differences in both the paths and intensity of influence on various child outcomes as a function of developmental stage. As the cognitive and academic needs of children change over time, the effects of parent work factors will no doubt change and must be examined.

We finish our discussion by exploring specific areas within the work-family literature that we would be well-served to integrate as we grow our efforts to employ comprehensive multidisciplinary approaches for understanding the impact of parent work factors on child cognitive and school outcomes. We also provide examples of research questions that exemplify the integration of work-family theory into Bronfenbrenner’s systems approach (See Table 1).

### 6.2. Role Balance and Family Routines

Research examining the intersection of parent work exosystem factors and child cognitive development would benefit from exploring the dynamics between parent work and family roles. Role theory (Biddle, 1986; Greenhaus & Beutell, 1985) provides insight into how domain-specific interactions, work conditions, and both work and family systems are pivotal for parents specifically. When there is conflict, roles in each domain are made more difficult, resulting in experiences of stress and strain (Bolger et al., 1989; Greenhaus & Beutell, 1985; Koeske & Koeske, 1993), and providing a context for negative spillover and crossover. Marks and MacDermid (1996) theorized that an individual’s composition of self was a composite system of roles and identities. They propose that individuals who balance their roles (regardless of their preferred structure) ultimately experience more well-being. Balance is characterized by equally engaging in all roles across role experiences to alleviate role overload and promote role ease (Marks & MacDermid, 1996). Role ease is characterized by the extent to which individuals feel that performing in each of their roles lacks difficulty. Therefore, if an individual can embody their role as a parent without any difficulty, meaning they can enter the role without issue and effectively perform behaviors that are consistent with the role, the parent role would have high levels of role ease. Moreover, role ease may support the establishment of family routines.

Parents have difficulty managing work and family roles when they lack routines and experience situational urgencies (Evans et al., 2005). Family routines may help working parents mitigate negative spillover and crossover, but the development of routines is not unaffected by parent work factors. For low-wage workers, schedule unpredictability and uncertainty of hours are just two factors that prevent the easy establishment of routines in the family domain. Established routines reflect a working parent’s recognition of how each of their roles fit together and knowledge of when and how to perform in each role effectively (i.e., role balance). Family routines reduce family conflict and improve child behavior (Hosokawa et al., 2023). Stability and predictability of parent work should therefore allow for the development and following of family routines. Working parents and their children may significantly benefit from resources that allow them to establish meaningful family routines (Ferretti & Bub, 2014). Conversely, individuals who lack established routines often find urgent situations to be problematic. For example, a low-wage parent cannot take on a project that requires extended work hours because they need to help their child with homework or have unreliable transportation. Low-income families are often less prepared for large-scale emergencies that impact the whole family (Mack et al., 2006) due in part to a parent’s work context that fails to support the development of routines.

Although informal social networks are often considered a meaningful resource for low-income families, they often lack consistency in availability and utility (Hill et al., 2021). Thus, the most frequently used resource for low-income parents may not help with routines or predictability. One approach for parents with extremely limited work resources has been to build a warm and supportive relationship with their children as a buffer of work and family stress (Bai & Repetti, 2015), supporting a quality over quantity perspective. Though this merely treats the symptoms, it is not the cause of stress.

### 6.3. Flexibility (Discretion and Control)

Flexible work arrangements, which include both formal and informal practices intended to provide workers with resources to manage their work and family responsibilities, have been explored for decades (Kossek & Michel, 2011). Though flexibility has long been identified as a tool to support employee wellbeing (French & Shockley, 2020; Gajendran & Harrison, 2007; Waples & Brock Baskin, 2021), the reality is more complex. In support of flexibility, Genadek and Hill (2017) found that mothers with flexible schedules or who work remotely spend more time with their children compared to mothers who do not have that form of work flexibility and control. Organizations that have flexibility built into their policies (i.e., flextime and flexplace) signal to employees their family-supportive organizational values (Thompson et al., 2015). Moreover, flexible work arrangements offer the potential for employees to better respond to demands in their work or family domains, reducing potential conflict and mitigating negative spillover and crossover effects. Congruent with broader schedule resources, maternity leave benefits are associated with healthier child development (Sattari et al., 2013). Even interventions that focus on parent involvement have a meaningful impact on school and non-school related outcomes for children (Cosso et al., 2022). Flextime availability has been related to increased parent–child time (Kim, 2020) and child sleep (Lee et al., 2019).

Flexibility has been highlighted as a resource for employees and especially for working parents. For low-income families, however, these benefits are elusive. Ballentine and Pilarz (2026) found that when parents have self-set flexible schedules, they are more likely to be involved with their child’s schooling. However, when employers set the schedule, they found no improvement in parents’ school involvement. Such findings reveal a disconnect between our broader understanding and assumptions about work schedule flexibility and its downstream impact on employees and their children. The impact of workplace flexibility, like many work-family support systems, may not generalize to low-wage workers (Agars & French, 2016; French & Agars, 2018). This is supported by growing evidence that flexibility for low-wage workers, rather than being a source of support, is more commonly an additional demand (French et al., 2024). Formal flextime and flexplace relationships, which tend to produce the most positive outcomes for working parents, are rarely available to low-wage workers (Kossek & Lautsch, 2018). For low-wage workers, job roles may be inconsistent with traditional flexibility assumptions (Lambert et al., 2012), and research suggests that rather than providing support, flexibility for low-wage workers often means uncertainty and unpredictability in hours and/or schedule (Agars & French, 2016; Henly & Lambert, 2014), which then becomes a significant demand to be managed by low-wage working parents.

For low-wage working parents, the demands of work uncertainty and the absence of formal organizational supports pose real threats to their ability to effectively manage the work-family interface. In response, many low-wage workers rely on informal solutions, often through coworker support, in order to create more functional flexibility (Lefrançois et al., 2017) or through drawing on non-work resources. Though, as Matthews et al. (2014) note, reliance on informal solutions and shuffling of resources may solve short-term problems but come at a cost (e.g., borrowing family time resources to alleviate work stress). Collectively, the literature on workplace flexibility illustrates the need to consider its formal and informal forms, whether flexibility is voluntary or non-voluntary, and the extent to which it creates real control and discretion for working parents. These variants and their implications for spillover and crossover offer fruitful questions for research on how low-wage work matters for child cognitive and academic outcomes.

### 6.4. Boundary Management

Boundary management refers to how individuals create, maintain, and negotiate boundaries around their work and family roles and responsibilities (Ashforth et al., 2000). Boundaries have both psychological and physical elements and are the points through which work and family intersect. Boundaries have discrete components, but individuals also vary in their preference for the extent to which boundaries allow integration across domains (Ammons, 2013). Boundary preferences range from fully integrated to fully segmented, and preferences exist across the continuum (Ammons, 2013; Kreiner, 2006). Although there is limited research on consistent boundary management strategies (Kossek et al., 2023), spillover and crossover effects may occur in part as a function of boundary integration but may also be dependent upon the congruence between boundary preferences and enactment.

Boundaries are the path through which spillover occurs (or not), suggesting that effective boundary management is a key mechanism explaining work factor effects on microsystem factors and child cognitive outcomes. Ammons (2013) found that boundary management strategies varied from strong boundaries protecting either work or family, to loose or nonexistent boundaries allowing for near complete integration. The desired and enacted boundary management strategies used may dictate how and when working parents can engage with their children and the quality of those interactions. When parents work longer hours, and their interactions with their children are more frequently disturbed (i.e., boundaries are permeable), they tend to have lower quality interactions and lower quality relationships with their children (Roeters et al., 2010).

The majority of boundary management research has focused on white-collar workers, and little is known about boundary management for low-wage workers and parents (Agars & French, 2016), but there is reason for concern. For white-collar workers, boundary integration may be a choice, whereas low-wage workers may have forced or involuntary integration. Additionally, low-wage employees may not have the resources available to have an extra room for a home office, or the freedom to schedule when they work (i.e., shift work, call work, etc.). Low-wage employees may be less likely to benefit from micro-transitions such as changing from “work” to “home” clothes when they get home, using their commute home as an opportunity to mentally change roles or decompress, or using means for reducing or eliminating work notifications from their electronic devices supported by role transitions (Ashforth et al., 2000; Nippert-Eng, 2008). These micro-transitions allow parents to better navigate their roles and both physically and psychologically transition more effectively from work to home or vice versa. Previously expressed concerns about lack of discretion and control for low-wage workers (Kossek & Lautsch, 2018), and lack of supportive policy (Lambert & Haley, 2021) are relevant here too, as they limit a low-wage working parent’s capacity to adjust boundary expectations and realities, and to respond to unexpected or unwanted boundary transitions.

More effective role transitions may be one mechanism for work-family enrichment or positive spillover and crossover. Eased role transitions and role ease (Marks & MacDermid, 1996) should foster positive parent–child interactions and facilitate parenting responsibilities. To increase role ease, employees may benefit from negotiating work schedules and clear boundaries to best manage work-family responsibilities (Kossek et al., 2006). To what extent, however, are these available to low-wage working parents? Boundary management represents one of the more promising areas of research in managing work-family conflict and negative spillover and crossover effects. For working parents, the ability to manage boundaries is a resource offering paths to manage current demands and protection from additional demands and their downstream effects on parent–child interactions and child cognitive and academic outcomes. For parents working low-wage positions, those promises are unclear but no less important.

### 6.5. Family Supportive Supervisor Behaviors

Supervisors as a source of support for working parents managing work and family responsibilities have been a growing interest (Crain & Stevens, 2018). In the context of Bioecological theory (Bronfenbrenner, 2005), leaders are a potentially powerful influence within the parent work exosystem. Accordingly, understanding the supportive role of supervisors, specifically Family Supportive Supervisor Behaviors (FSSB; Hammer et al., 2011), has been a promising and growing area in the work-family literature. FSSBs consist of four dimensions, including emotional support, instrumental support, role modeling, and creative work-family management. Emotional support is the care or concern shown by the supervisor in consideration of an employee’s affect, particularly in relation to work and family roles. Instrumental support is the utilization of organizational policy and practices to allot employees with more resources, such as flexibility and decision-making power. Role modeling is provided when supervisors model behaviors that they use to balance their own work and family needs. Lastly, creative work-family management occurs when supervisors proactively develop solutions for employees more broadly in support of work-family needs (Hammer et al., 2011).

There is growing evidence that employee experiences of FSSBs relate to reduced work-family conflict (Hammer et al., 2013; Michel et al., 2011) and reductions in stress and exhaustion (Koch & Binnewies, 2015). FSSBs are related to reduced work-family conflict and improved psychological well-being (Michel et al., 2011). FSSBs are positively related to work-family balance and enrichment (Hammer et al., 2009; Odle-Dusseau et al., 2012). Moreover, FSSBs are an area where spillover and crossover effects on children have been considered. Research has found that FSSBs are related to parents’ time spent with children (Davis et al., 2015; McHale et al., 2016), children’s sleep quality (McHale et al., 2015), and children’s affective well-being (Lawson et al., 2016). This is a promising start for advancing work that captures the influence of supervisors as a parent work factor on child cognitive and school outcomes. Critically, we must also consider what this might look like for low-wage working parents.

The lack of formal work-family policies and the increased schedule uncertainty and work demands that typically accompany low-wage jobs suggest FSSBs may serve as an even more critical source of support for working parents managing spillover and crossover of stress and strain from work into the family unit and onto children. Pajic et al. (2021) state that low-SES parents are likely to benefit more from supportive leadership due to less job autonomy or resources compared to those in higher-paying or higher-SES situations (e.g., equitable pay, role clarity, education, and training). Questions remain, however, about what FSSBs look like in the context of low-wage work. Studies have demonstrated that supervisors can be successfully trained on FSSBs (Hammer et al., 2011; Hammer et al., 2016), but Ellis et al. (2022) note that many supervisors do not believe or are unsure that FSSBs are considered a part of their job. Moreover, supervisors of low-wage workers often occupy positions that lack the control and discretion necessary to provide support. While FSSBs are a promising exosystem factor to consider for their impact on child cognitive and school outcomes, research to date argues both for their heightened importance for low-wage workers and that their availability may be unlikely.

### 6.6. Approaches to Consider for Future Work

Utilizing an integrated theoretical approach to advancing our understanding of the work-family interface for low-wage workers and their families would also benefit from thoughtful integration of recent methodological advances in the study of work–life factors, and from more targeted utilization of methods specifically appropriate for capturing the challenging landscape of low-wage work and family life. Given the lack of schedule control, limited flexibility, and the heightened challenges with family transitions (e.g., child handoffs), more careful consideration of methodologies that incorporate time-based elements would be beneficial (see Allen et al., 2019 for an overview). Moreover, French and Allen (2020) illustrated the importance of capturing individual episodes of work-family conflict. For low-wage worker families, which often exist in under-resourced environments, individual episodes of conflict are likely to be heightened, underscoring the need for taking an episodic approach. Finally, though cumbersome, an increased use of daily diary methodologies is needed as they offer the opportunity to capture time-based phenomena and to examine in depth the work-family interface experienced by low-wage workers and their families (c.f., Brenning et al., 2023; Radcliffe et al., 2023).

## 7. Conclusions

Our intention was to illustrate how efforts to understand child cognitive and school outcomes would benefit from increased consideration of research advancements and theory from the work and family interface. We provide a multidisciplinary systems approach based on Bronfenbrenner’s Ecological systems framework, integrating Job Demands-Resources theory, role theory, and spillover-crossover theory, to highlight that parent work is not isolated in parent experiences but serves as a critical exosystem factor that impacts the family systems, parent–child interactions, and ultimately child development. We explored evidence that suggests that job demands, schedule (in)stability, (weak) boundary control/management, and (limited) organizational support may determine and more specifically undermine parent–child interactions and routines. Conversely, resources such as flexibility, family-supportive supervisor behaviors, and role clarity may foster more work-family enrichment or buffer work-family conflict and ultimately child cognitive and academic outcomes/development. Critically, these highlighted processes operate across systems that are particularly consequential for low-wage and resource-limited families, as they often have limited access to supportive policies and practices. Furthermore, advancing research and practice on child cognitive and school outcomes will benefit from a multilevel, multidisciplinary approach that moves beyond helping parents cope (the symptom) and toward developing systemic conditions that shape the work, family, and family system interactions in the first place (the problem). Of final note, it would be disingenuous to suggest these advances can occur without industry and/or policy-level system changes. We hope our call inspires research that will inform advanced legislation on predictive scheduling, parental leave, and employer responsibilities to worker families and well-being.

## Figures and Tables

**Table 1:** Research recommendations integrating work-family theories and ecological systems.

System	Relevant Work-Family Theories	Sample Research Questions
Microsystem	Role Balance	How do parents’ role expectations around work and family impact engagement in children’s school activities?
	Job Demands-Resources	What family resources help low-wage working parents balance demands from unpredictable work schedules that limit parent–child interactions?
	Boundary Management	In what ways do segmentation and integration strategies differentially affect parental capacity for high-quality parent–child interactions?
	Spillover-Crossover	How does spousal support reduce crossover of stress from working parents to children?
Mesosystem	Role Balance	How does work role stress manifest for parents when work and the child’s school expectations are incompatible?
	Job Demands-Resources	How do clear work and family role expectations impact parent utilization of work resources (e.g., flextime) that support parent engagement in their child’s school activities?
	Boundary Management	Does workplace support of employee segmentation preferences impact parent engagement and child academic success?
	Spillover-Crossover	How does schedule inflexibility and uncertainty impact the development of family routines, and in what ways do family factors impact that process in functional and dysfunctional ways?
Exosystem	Role Balance	How do rigid expectations around work availability impact children’s academic performance?
	Job Demands-Resources	What work demand factors are most related to stress and strain for working parents in low-wage jobs?
	Boundary Management	How do organizational norms for parent availability predict different child cognitive and academic outcomes?
	Spillover-Crossover	What is the impact of non-voluntary flexibility on stress for parents of a child with special needs?
	Family Supportive Supervisor Behaviors	What does family supervisor support look like for low-wage working parents?

## Data Availability

No new data were created or analyzed in this study. Data sharing is not applicable to this article.

## Note

1 A model could incorporate broader factors such as societal norms around work or economic factors, identified by Bronfenbrenner (1994) as macrosystems. For the sake of clarity and manageability, we have elected to stop our integration at theories addressing exosystem and mesosystem factors.
